# Editorial: Immunometabolism applied to exercise, nutrition and pharmacology treatment

**DOI:** 10.3389/fimmu.2023.1360040

**Published:** 2024-01-12

**Authors:** Fábio Santos Lira, José Cesar Rosa-Neto, James Edward Turner

**Affiliations:** ^1^ Exercise and Immunometabolism Research Group, Post-graduation Program in Movement Sciences, Department of Physical Education, Universidade Estadual Paulista (UNESP), São Paulo, Brazil; ^2^ Centro de Investigação em Desporto e Atividade Física (CIDAF), University of Coimbra, Coimbra, Portugal; ^3^ Department of Cell and Developmental Biology, Immunometabolism Research Group, Institute of Biomedical Sciences, University of Sao Paulo (USP), Sao Paulo, Brazil; ^4^ School of Sport, Exercise and Rehabilitation Sciences, University of Birmingham, Birmingham, United Kingdom

**Keywords:** immunometabolism, exercise, nutrition, immune cells, pharmacology

## Introduction

Examining the energetic metabolism of immune cells is not a recent development (1) but the field of ‘immunometabolism’ has rapidly expanded over the past decade (2). A growing area within immunometabolism examines the influence nutrients and energetic substrates have in regulating the functions of immune cells, often in the context of metabolic disruption and systemic inflammation. Ageing and several chronic conditions, including obesity, type 2 diabetes, dyslipidemia and auto-immune inflammatory diseases require further investigation to examine how immunometabolism influences pathophysiology and the molecular mechanisms governing cell metabolism. Exercise and nutrition interventions are commonly employed to improve metabolic and inflammatory profiles and to counter the pathophysiology of chronic disease, but interaction with immunometabolism is understudied. The present Research Topic, includes immunometabolism studies undertaken in the context of exercise, nutrition and pharmacological treatment of chronic inflammatory disease.

Studies have shown that eating an olive oil-containing Mediterranean diet – comprising high levels of mono-unsaturated fatty acids – is associated with a variety of health benefits. In this Research Topic, Döding et al. compared the impact of a mono-unsaturated fatty acid enriched diet to a saturated fatty acid enriched diet on bone metabolism, bone microarchitecture and inflammation in the context of experimental *P. gingivalis* infection in mice. A saturated fatty acid enriched diet reduced systemic bone microarchitecture and increased lipotoxic metabolites but these changes could be reversed by a mono-unsaturated fatty acid enriched diet.

In the setting of pharmacological treatment of chronic inflammatory disease, Peng et al. demonstrated using single-cell sequencing that upregulation of ascorbate and aldarate metabolism, as well as fatty acid degradation, enhances the immunosuppressive capacity of regulatory T cells in patients with psoriatic arthritis and limits the pro-inflammatory action of central memory and effector memory T cells. The authors suggest that ascorbic acid and fatty acid metabolic pathways may be an adjunctive reprogramming strategy with adalimumab and etanercept –biological therapies targeting tumour necrosis factor-alpha (TNF-α).

It is known that both redox responses to exercise bouts and metabolic responses to glucose ingestion exhibit individual variation. Thomas et al. combine this knowledge to examine whether responses to acute high glucose ingestion are influenced by a bout of exercise performed 3 or 24 hours before. Several oxidative stress biomarkers and antioxidant measurements increased immediately after exercise, returning towards pre-exercise values within 3 hours. Redox responses to glucose ingestion depended on the biomarker and timepoint examined. The magnitude and direction of change was variable between individuals and the pattern of change was partly influenced by the timing of exercise before glucose ingestion. Thus, this study highlights the importance of presenting individual data and controlling for exercise in the hours or days prior to redox and metabolic measurements.

**Figure 1:**
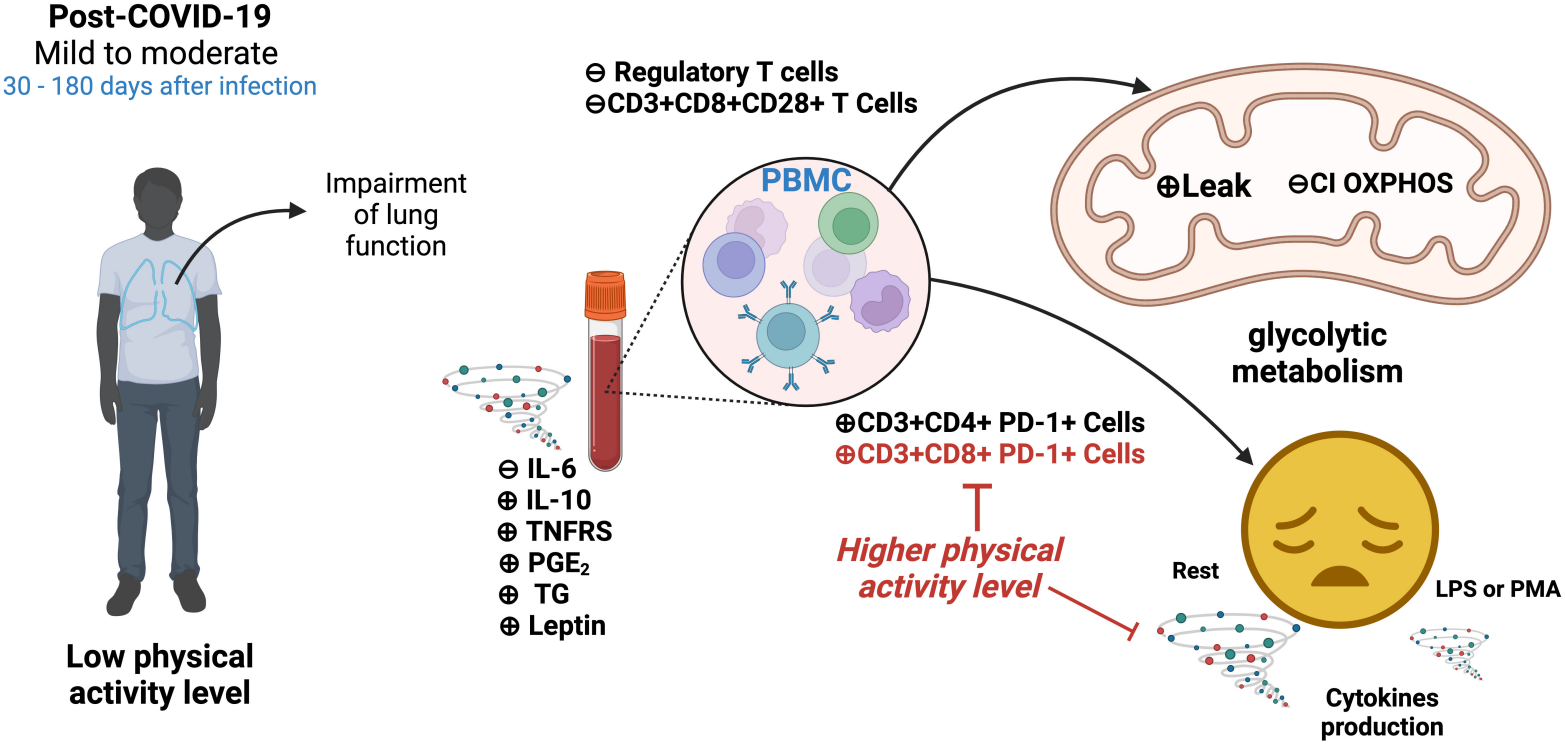
Young people post-infected with SARS-CoV-2 exhibited an exhaustive profile and anaerobic metabolism in PBMCs, and Physical Activity Level protects intracellular changes partially. IL-6, Interleukin 6; IL-10, Interleukin 10; TNFRS, Soluble TNF-α receptor; PGE_2_, Prostaglandin E2; TG, Triglyceride; PBMC, Peripheral blood mononuclear cell; T Cells, T lymphocytes; PD-1, Programmed death protein-1; Leak, Complex I respiration mitochondrial; CI OXPHOS; Complex I Oxidative phosphorylation respiration mitochondrial; LPS, Lipopolysaccharide; PMA, Phorbol 12-myristate 13-acetate; ⊕, Increased; ⊖, Decreased. Red text indicates a protective effect of physical activity.

Finally, Silva et al. explored the potential influence of physical activity levels on systemic and cellular immunometabolic measurements in young adults after mild-to-moderate COVID-19. Higher physical activity was partially associated with better metabolic and inflammatory profiles in the serum of post-COVID-19 patients. In addition, higher physical activity was associated lower expression of PD-1 in CD8+ T cells and lower TNF-α and IL-6 production by LPS- and PMA-stimulated peripheral blood mononuclear cells. As shown in **Figure 1**, the yellow face represents exhausted immune cells, which might be related to hyperactivation during COVID-19, leading to dysregulated cytokine production. The cyclones next to the image of PBMCs and tube of blood represent higher/lower cytokine production by PBMCs or higher/lower cytokines in serum. These changes were partially protected by Physical Activity Level.

Taken together, this Research Topic highlights important interaction between nutrients, physical activity, exercise and pharmacological treatment of inflammatory disease with systemic and cellular immunometabolic profiles.

## Author contributions

FL: Writing – original draft, Writing – review & editing. JR-N: Writing – original draft, Writing – review & editing. JT: Writing – original draft, Writing – review & editing.
